# WDR79 promotes the proliferation of non-small cell lung cancer cells via USP7-mediated regulation of the Mdm2-p53 pathway

**DOI:** 10.1038/cddis.2017.162

**Published:** 2017-04-13

**Authors:** Yang Sun, Lanqin Cao, Xunan Sheng, Jieying Chen, Yu Zhou, Chao Yang, Tanggang Deng, Hongchang Ma, Peifu Feng, Jing Liu, Weihong Tan, Mao Ye

**Affiliations:** 1 Molecular Science and Biomedicine Laboratory, State Key Laboratory for Chemo/Biosensing and Chemometrics, College of Biology, College of Chemistry and Chemical Engineering, Collaborative Innovation Center for Molecular Engineering for Theranostics, Hunan University, Changsha, Hunan 410082, China; 2 Department of Gynecology, Xiangya Hospital, Central South University, Changsha, Hunan 410078, China; 3 College of Life and Environmental Sciences, Gannan Normal University, Ganzhou, Jiangxi 341000, China; 4 School of Life Sciences, State Key Laboratory of Medical Genetics, Central South University, Changsha, Hunan 410078, China

## Abstract

WD repeat protein 79 (WDR79) is a member of the WD-repeat protein family and functions as a scaffold protein during telomerase assembly, Cajal body formation and DNA double strand break repair. We have previously shown that WDR79 is frequently overexpressed in cell lines and tissues derived from non-small cell lung cancer (NSCLC) and it accelerates cell proliferation in NSCLC. However, the detailed mechanism underlying the role of WDR79 in the proliferation of NSCLC cells remains unclear. Here, we report the discovery of a molecular interaction between WDR79 and USP7 and show its functional significance in linking the Mdm2-p53 pathway to the proliferation of NSCLC cells. We found that WDR79 colocalized and interacted with USP7 in the nucleus of NSCLC cells. This event, in turn, reduced the ubiquitination of Mdm2 and p53, thereby increasing the stability and extending the half-life of the two proteins. We further found that the functional effects of WDR79 depended upon USP7, because the knockdown of USP7 resulted in their attenuation. Finally, we demonstrated that WDR79 promoted the proliferation of NSCLC cells via USP7. Taken together, our findings reveal a novel molecular function of WDR79 and may lead to broadly applicable and innovative therapeutic avenues for NSCLC.

The *WDR79* gene on chromosome 17p13 encodes both an antisense transcript (WRAP53α) for p53 stabilization^1^ and a protein (WDR79, WRAP53, WRAP53β or TCAB1) with six individual WD-repeat domains via the use of alternative transcriptional start sites. The protein WDR79 functions as a scaffold protein that is involved in telomerase localization, telomere and Cajal body assembly, and DNA double strand break repair.^2, 3, 4, 5, 6, 7, 8^ The dysfunction of WDR79 has been implicated in human diseases. Specifically, germline mutations in WDR79 that affect the WD-repeat domain result in congenital dyskeratosis,^9^ and WDR79 overexpression has been observed in rectal cancer,^10^ head and neck carcinomas,^11^ squamous cell carcinoma,^12^ breast cancer^13^ and ovarian cancer.^14^ In a previous study, we also found that WDR79 is frequently overexpressed in cell lines and tissues derived from non-small cell lung cancer (NSCLC) and accelerates NSCLC cell proliferation.^15^ However, the detailed mechanism underlying the effect of WDR79 on the proliferation of NSCLC cells remains unclear.

Ubiquitin-specific proteases (USPs) constitute the largest subfamily of deubiquitinases, which consist of more than 60 members. They function as cysteine proteases to remove ubiquitin from specific protein substrates and allow protein salvage from proteasomal degradation or regulate their subcellular location and activation. USP7, a member of the USP family, is first defined as Herpes virus associated cellular factor (HAUSP) and is critical for genome stability, epigenetic regulation, cell cycle, apoptosis, viral infection immunity and stem cell maintenance.^16, 17, 18, 19, 20^ Clinically, USP7 is reportedly associated with tumorigenesis, including prostate cancer, multiple myeloma cancer, ovarian cancer, breast cancer and NSCLC.^20, 21, 22, 23, 24^

As a tumour suppressor protein, p53 function is abnormal in over 50% of human cancers.^25, 26^ In normal unstressed cells, p53 is a very unstable protein with a short half-life and maintained at a low level due to its rapid degradation via the ubiquitin-dependent proteasome pathway.^27, 28^ However, in response to diverse cellular stress, such as DNA damage, hypoxia, telomeres shortening and oncogene activation, p53 is rapidly stabilized because its degradation is blocked, which results in cell cycle arrest, apoptosis and cellular senescence.^29^ Mdm2 is the E3 ubiquitin ligase of p53 and binds to p53 to promote its degradation.^30^ Recent studies have revealed that USP7 is involved in the regulation of the p53-Mdm2 pathway. USP7 can bind Mdm2 or p53 via its N-terminal and C-terminal regions in a mutually exclusive manner, which consequently stabilizes the two proteins by removing ubiquitin. The *in vitro* binding affinity of USP7 for Mdm2 is several-fold higher than for p53, which implies that Mdm2 is the preferred substrate of USP7 in normal cellular homeostasis. In addition, USP7 may deubiquitinate p53 via Mdm2.^31^ However, upon DNA damage, ataxia telangiectasia mutated-dependent phosphorylation of Mdm2 decreased the binding affinity between USP7 and Mdm2, tilting the balance towards p53 stabilization.^31, 32, 33, 34^

In this study, we report a new molecular process in which WDR79 interacts with USP7 to modulate the stability of Mdm2 and p53, which promotes the proliferation of NSCLC cells. In particular, we found that WDR79 colocalized and interacted with USP7 in the nucleus of NSCLC cells. This event, in turn, increased the stability and prolonged the half-life of Mdm2 and p53 by decreasing ubiquitination. We further found that the functional effects of WDR79 depended on USP7 and manifest a net outcome as promoting the proliferation of NSCLC cells. Taken together, our findings reveal a novel role of WDR79 in the proliferation of NSCLC cells and could pinpoint a new mechanism by which WDR79 and USP7 functionally interact to modulate the Mdm2-p53 pathway.

## Results

### WDR79 interacts with USP7

**Figure 1:**
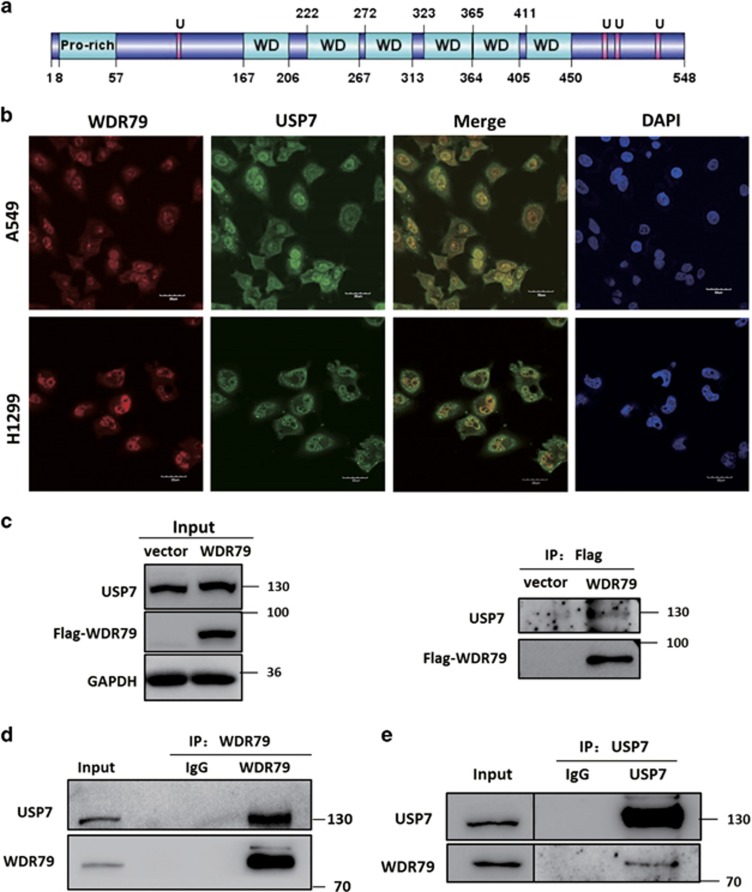
WDR79 interacts with USP7. (**a**) The predicted functional domains of WDR79 are shown (U: USP7 binding site). (**b**) A549 and H1299 cells were fixed and incubated with anti-WDR79 and anti-USP7 antibodies, followed by staining with DyLight 594 or Dylight 488-conjugated IgG. DAPI was used for nuclei staining (bars, 30 μm). (**c-e**) A549 cells transfected with the indicated constructs or H1299 cells were lysed and lysates were subject to immunoprecipitation with anti-Flag (**c**), anti-WDR79 (**d**), or anti-USP7 (**e**) antibodies. The immunoprecipitates were then blotted with the indicated antibodies

Our previous studies have shown that WDR79 is mainly located in the nuclei in NSCLC tissues and cell lines. Because of the limited knowledge regarding WDR79 function, we first characterized the functional domains of WDR79 by ELM (Eukaryotic Linear Motif) to get a clue on WDR79 function in nuclei. Four putative USP7 binding domains were identified in WDR79, one was located at the N terminus, and three at the C terminus (Figure 1a). To test the hypothesis that WDR79 is associated with USP7, we first examined the subcellular localization of WDR79 and USP7 by indirect immunofluorescence. Endogenous WDR79 and USP7 were labelled with their corresponding antibodies conjugated to DyLight 594 and 488, respectively. An intense yellow colour was observed in merged images of USP7 with WDR79 expression (Figure 1b), indicating that WDR79 and USP7 colocalized in the nucleus. To confirm that WDR79 indeed interacts with USP7 *in vivo*, Flag-WDR79 plasmid was transfected into A549 cells (p53^+/+^). We found that USP7 was co-immunoprecipitated by anti-Flag antibody in WDR79-overexpressing cells but not in negative control cells transfected with the same amount of empty vector (Figure 1c). Next, the endogenous interaction between USP7 and WDR79 was also investigated by co-immunoprecipitation. As shown in Figures 1d and e, USP7 was detected in the anti-WDR79 immunoprecipitates from H1299 cells (p53^-/-^) and vice versa, but not in an isotype-matched negative control IgG. Collectively, these results indicate that WDR79 interacts with USP7 and that their interaction is independent of p53 expression.

### WDR79 influences the protein level of Mdm2 and p53 but not their localization

**Figure 2:**
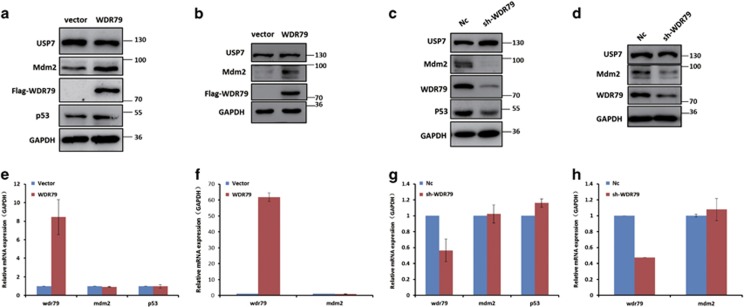
WDR79 influences the protein levels of Mdm2 and p53. (**a** and **b**) A549 (**a**) and H1299 (**b**) cells transfected with the indicated constructs for 24 h. Proteins in lysates were detected by western blotting using the indicated antibodies. (**c** and **d**) A549 (**c**) and H1299 (**d**) cells infected with lentivirus encoding the indicated shRNA were lysed and lysates were blotted with the indicated antibodies. (**e** and **f**) A549 (**e**) and H1299 (**f**) cells were transfected with the indicated constructs. Twenty-four hours later, the indicated mRNA was extracted and subjected to real-time PCR. (**g** and **h**) A549 (**g**) and H1299 (**h**) cells were infected with lentivirus encoding the indicated shRNA. Twenty-four hours later, the indicated mRNA was extracted and subjected to real-time PCR

**Figure 3:**
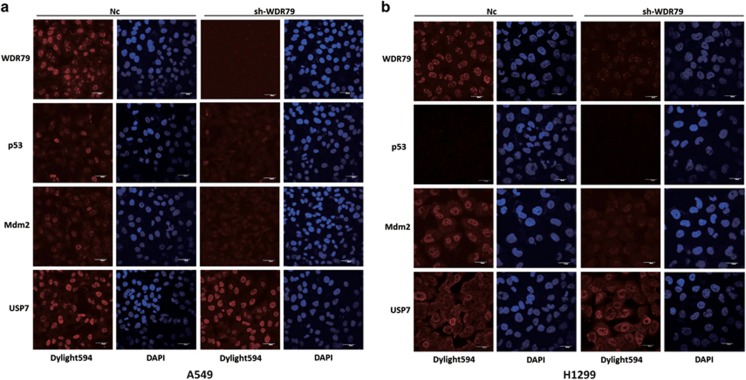
WDR79 does not regulate the subcellular location of p53, Mdm2 and USP7. (**a** and **b**) A549 cells (**a**) and H1299 cells (**b**) infected with lentivirus encoding the indicated shRNA were fixed and stained as the indicated. DAPI was used for nuclei staining. Scale bars represent 30 μm

USP7 is a key deubiquitinase involved in the regulation of the p53-Mdm2 pathway and its forced expression results in p53 and Mdm2 stabilization.^18, 34, 35, 36, 37^ Given the interaction between WDR79 and USP7 described above, we investigated the effect of WDR79 on the protein levels of Mdm2, p53 and USP7. To this end, we first overexpressed vector control and Flag-WDR79 in A549 cells and examined protein expression by western blotting. The results show that WDR79 overexpression significantly increases the protein levels of Mdm2 and p53 compared with the control (Figure 2a). Notably, the expression of Mdm2 is well known to be primarily controlled by p53.^38, 39^ To exclude the effect of p53 on Mdm2 expression, we also performed the assay in p53-null H1299 cells and found that the protein levels of Mdm2 were still increased after Flag-WDR79 overexpression (Figure 2b), suggesting that the upregulation of Mdm2 by WDR79 is independent of p53 expression. Next, we measured the expression levels of Mdm2 and/or p53 in both A549 and H1299 cells whose endogenous WDR79 were knocked down using WDR79-specific short hairpin RNA (shRNA). As shown in Figures 2c and d, knockdown of WDR79 led to a decrease in Mdm2 and/r p53 levels. However, changes in WDR79 expression did not appear to affect USP7 levels. To determine whether the regulation of Mdm2 and p53 by WDR79 is mediated at the level of gene transcription, we examined the mRNA levels of Mdm2 and p53 by real-time PCR after downregulation or overexpression of WDR79. We found that the levels of Mdm2 and p53 mRNA were not significantly affected by WDR79 (Figures 2e–h), indicating that the effect of WDR79 on the Mdm2 and p53 levels is not due to changes in their transcription, but more likely occurs at the posttranslational level (protein level). Furthermore, the protein levels and cellular localization of Mdm2, p53 and USP7 were further detected by immunofluorescent staining after WDR79 was knocked down in A549 and H1299 cells. As shown in Figure 3, the knockdown of WDR79 resulted in a weak fluorescent staining of Mdm2 and p53, but not USP7, which is consistent with the result that WDR79 downregulation significantly diminished endogenous levels of Mdm2 and p53. Meanwhile, the knockdown of WDR79 did not seem to change the cellular localization of Mdm2, p53 and USP7 compared to control A549 and H1299 cells (Figures 3a and b).

### WDR79 stabilizes Mdm2 and p53 by protecting them from proteasome-mediated degradation

**Figure 4:**
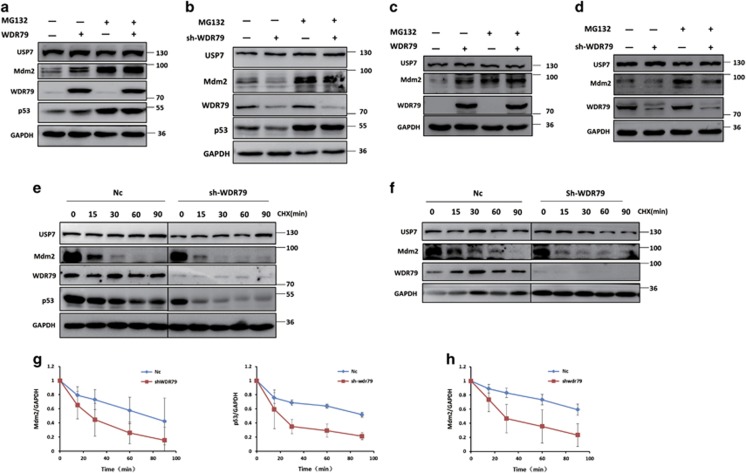
WDR79 stabilizes Mdm2 and p53 by protecting them from proteasome-mediated degradation. (**a** and **b**) A549 cells transfected with the indicated constructs (**a**) or infected with lentivirus encoding the indicated shRNA (**b**) were treated with DMSO or MG132 for 6 h. Expression of the indicated protein was examined by western blotting using the indicated antibodies. (**c** and **d**) H1299 cells transfected with the indicated constructs (**c**) or infected with lentivirus encoding the indicated shRNA (**d**) were treated with DMSO or MG132 for 6 h. Expression of the indicated protein was examined by western blotting using the indicated antibodies. (**e** and **f**) A549 (**e**) and H1299 (**f**) cells infected with lentivirus encoding the indicated shRNA were treated with cycloheximide (50 μg/ml), harvested at the indicated point and then immunoblotted with the indicated antibodies. (**g** and **h**) Quantification of the p53 or Mdm2 protein levels relative to GAPDH. Intensities of blots were analysed by the ImageJ software

Approximately 80% of intracellular proteins are degraded by the ubiquitin-proteasome system (UPS). To elucidate the mechanism by which WDR79 regulates the protein levels of Mdm2 and p53, A549 and H1299 cells were treated with the proteasome inhibitor MG132. As expected, in the absence of MG132, changes in the Mdm2 and p53 protein levels were accompanied by WDR79 overexpression or downregulation in A549 cells. However, MG132 treatment stabilized and eventually attenuated the changes caused by WDR79 (Figures 4a and b). Similar results were also obtained in H1299 cells (Figures 4c and d), suggesting that WDR79 regulates Mdm2 and p53 levels in a proteasome-dependent manner.

Both Mdm2 and p53 have a short half-life and are strictly maintained at low levels by ubiquitin-mediated proteolytic degradation in unstressed cells.^40, 41^ To assess the ability of WDR79 to regulate Mdm2 and p53 stability, A549 and H1299 cells with or without WDR79 knockdown were treated with cycloheximide to inhibit protein biosynthesis. Cell lysates collected at the indicated time points were analysed by western blotting, which revealed that the half-lives of Mdm2 and p53 were significantly shorter in WDR79-knockdown cells than in control cells (Figures 4e–h). The above results demonstrate that WDR79 stabilizes Mdm2 and p53 by preventing their proteasomal degradation.

### WDR79 is involved in the regulation of the ubiquitination of Mdm2 and p53

**Figure 5:**
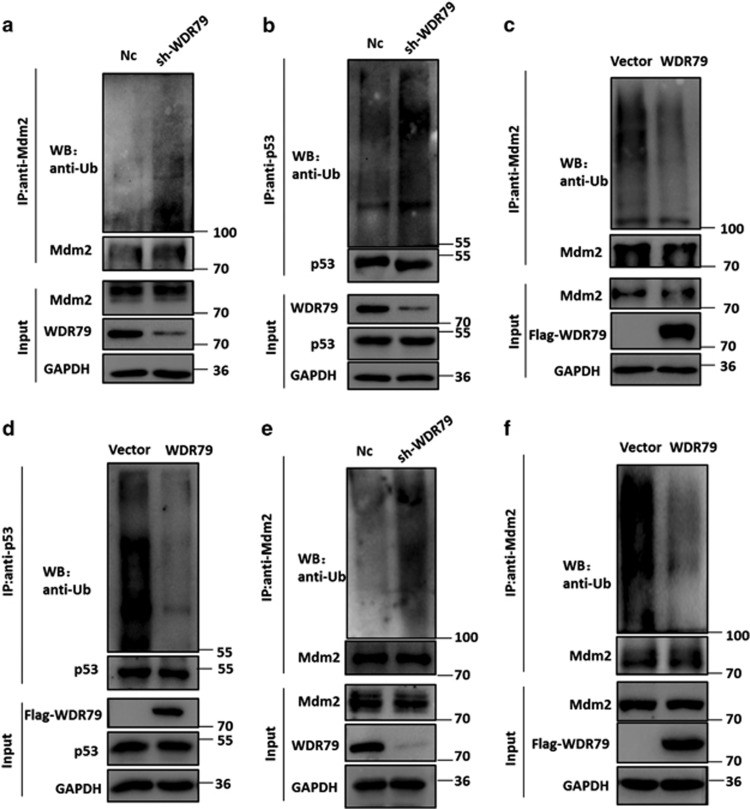
WDR79 affects the ubiquitination of Mdm2 and p53. (**a** and **b**) A549 cells infected with lentivirus encoding the indicated shRNA were treated with MG132 for 6 h. Lysates were immunoprecipitated with anti-Mdm2 (**a**) or anti-p53 (**b**) antibody. The ubiquitination of the Mdm2 and p53 was analysed by western blotting using anti-ubiquitin antibody. (**c** and **d**) A549 cells transfected with the indicated constructs were treated with MG132 for 6 h. The ubiquitination of Mdm2 and p53 was analysed as above. (**e** and **f**) H1299 cells infected with lentivirus encoding the indicated shRNA (**e**) or transfected with the indicated constructs (**f**) were treated with MG132 for 6 h. The ubiquitination of Mdm2 and p53 was analysed as above

Mdm2 is a key negative regulator of p53 via its E3 ligase activity and itself subject to auto-ubiquitination.^42, 43^ Because WDR79 influences the half-lives of Mdm2 and p53, we hypothesized that WDR79 may be involved in the regulation of their ubiquitination. To test this hypothesis, cells were treated with the proteasome inhibitor MG132 such that polyubiquitinated Mdm2 and p53 proteins were accumulated for detection. As shown in Figures 5a−d, Mdm2 and p53 ubiquitination was significantly increased when WDR79 was knocked down, whereas the overexpression of WDR79 decreased their ubiquitination in A549 cells. Similar results were obtained in H1299 cells (Figures 5e and f). Taken together, these findings suggest that WDR79 controls the stability of Mdm2 and p53 by modulating their ubiquitination prior to proteasomal degradation.

### WDR79 mediates Mdm2 and p53 stabilization in a UPS7-dependent manner

**Figure 6:**
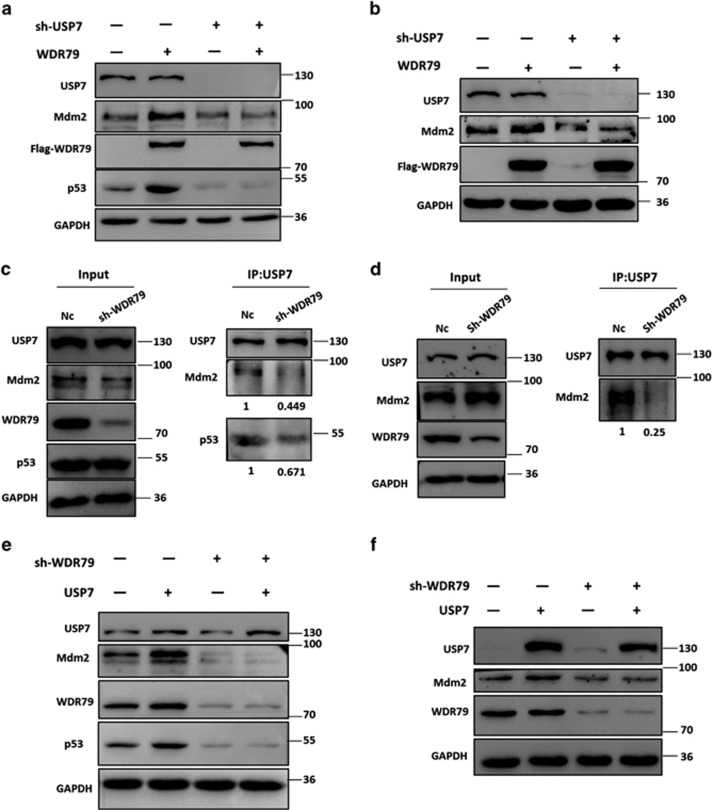
WDR79 mediates Mdm2 and p53 stabilization in a UPS7-dependent manner. (**a** and **b**) A549 (**a**) or H1299 (**b**) cells infected with lentivirus encoding the indicated shRNA were transfected with the indicated constructs. Lysates were blotted with the indicated antibodies. (**c** and **d**). A549 (**c**) or H1299 (**d**) cells infected with the lentivirus encoding the indicated shRNA were treated with MG132 for 6 h. Immunoprecipitation were performed with anti-USP7 antibody. The immunoprecipitates were then blotted with the indicated antibodies. (**e** and **f**) A549 (**e**) or H1299 (**f**) cells infected with lentivirus encoding the indicated shRNA were transfected with the indicated constructs. Lysates were blotted with the indicated antibodies

WDR79 is a scaffold protein not known to possess any enzymatic activity. Because it interacts with and exerts effects similar to that of USP7 on Mdm2 and p53, we surmised that WDR79 acts via the deubiquitinating activity of USP7. To test this hypothesis, USP7 was knocked down with shRNA. As shown in Figures 6a and b, knockdown of USP7 reduced the levels of Mdm2 and p53, a result consistent with that of a previous study. Moreover, the data revealed that knockdown of USP7 attenuated the stabilizing effect of WDR79 on Mdm2 and p53.

Scaffold proteins have been shown to promote cellular protein assembly. To assess the effect of WDR79 on the assembly of USP7 with its substrates, endogenous WDR79 was knocked down in A549 and H1299 cells. We then employed co-immunoprecipitation to identify possible changes in the binding of USP7 to Mdm2 and p53. We found that the USP7-Mdm2 and USP7-p53 interactions were significantly reduced in WDR79 shRNA-treated cells compared with control cells (Figures 6c and d). Intriguingly, WDR79 knockdown seemed to have a more profound impact on the interaction between USP7 and Mdm2 than the interaction between USP7 and p53. We inferred that this difference could be due to USP7 having more extensive interactions with Mdm2 than p53 does under physiological conditions.

Because WDR79 affected the interaction of USP7 with Mdm2 and p53, we hypothesized that WDR79 is involved in USP7-mediated regulation of Mdm2 and p53. To test this hypothesis, Flag-USP7 was transfected into WDR79-knockdown cells. As expected, the effect of USP7 on Mdm2 and p53 markedly decreased after WDR79 was knocked down (Figures 6e and f), suggesting that WDR79 plays an important role in USP7-mediated regulation of Mdm2 and p53. Overall, these data indicate that WDR79 and USP7 are close partners in the regulation of Mdm2 and p53.

### WDR79 promotes NSCLC cell proliferation through USP7

**Figure 7:**
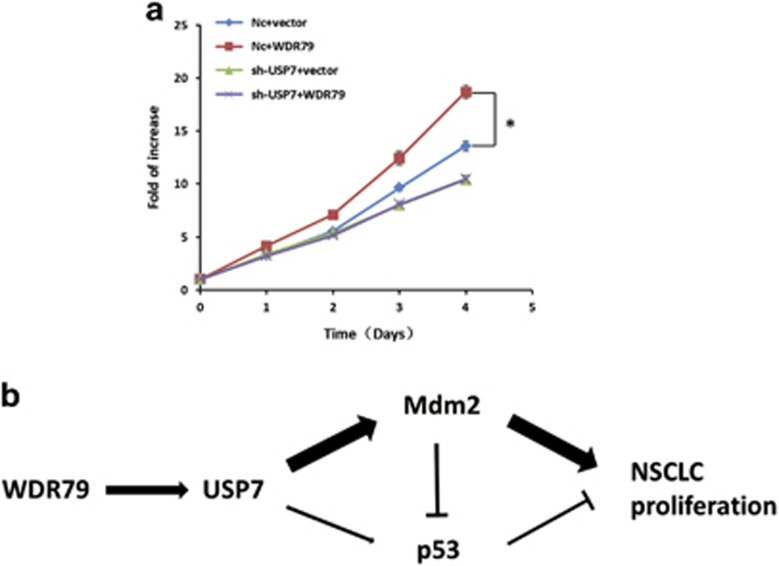
WDR79 promotes NSCLC cell proliferation via USP7. **(a**) A549 cells infected with lentivirus encoding the indicated shRNA were transfected with the indicated constructs. Cell proliferation was analysed with MTT at the indicated time points. (*n*=3; *P<0.05). (**b**) A schematic illustration of the working model that WDR79 promotes the proliferation of NSCLC cells via USP7-mediated regulation of the Mdm2-p53 pathway

We have previously shown that WDR79 is frequently overexpressed in cell lines and tissues derived from NSCLC and it accelerates cell proliferation in NSCLC cell lines. To identify the necessity of USP7 for the growth-promoting effect of WDR79, we analysed cell growth using MTT assay. We found that WDR79 overexpression promoted cell proliferation compared with control cells, which is consistent with our previous results. However, USP7 downregulation effectively attenuated the growth-promoting effect of WDR79 (Figure 7a), indicating that WDR79-mediated cell growth depends on USP7.

## Discussion

The *WDR79* gene encodes two functional products via the use of alternative transcription start sites: one is an antisense transcript (WRAP53α) that stabilizes p53 by binding to the 5′UTR of p53 mRNA, the other is a protein (WDR79, WRAP53, WRAP53β or TCAB1) with six individual WD-repeat domains that functions as a scaffold protein involved in the maintenance of Cajal bodies, telomere elongation and DNA repair. Previous studies have shown that WDR79 (WRAP53β) protein cannot regulate p53 expression.^44^ Intriguingly, we found although WDR79 protein did not affect the transcription of p53 mRNA, it could stabilize p53 protein by interacting with deubiquitinating enzyme USP7 via the ubiquitin proteasome system in the A549 NSCLC cell line. We provide several lines of evidence to support this conclusion. First, the overexpression of full-length WDR79 protein without overlap sequences with p53 mRNA can increase p53 level. Second, neither the overexpression nor the knockdown of WDR79 influenced the transcription of p53 mRNA. Third, the proteasome inhibitor MG132 abolished the effect of WDR79 on p53, indicating that WDR79 regulates protein level of p53. Fourth, WDR79 affected p53 protein stability in a half-life assay using the protein synthesis inhibitor cycloheximide. Fifth, WDR79 interacts with USP7. Moreover, knockdown of USP7 attenuated the effect of WDR79 on p53, revealing that WDR79 acts as a scaffold protein to affect p53 via USP7.

The WD-repeat domain is defined by four or more repeating units that contain a conserved core of 40–60 amino acids that begins with a glycine-histidine dipeptide and ends with a tryptophan-aspartic acid (WD) dipeptide.^45^ The human genome encodes ~300 proteins containing WD-repeat domains that are thought to fold into β-propeller structures and form a platform without any catalytic activity for the assembly of multiple protein complexes.^46^ Many studies have revealed that WD-repeat proteins are involved in ubiquitin-proteasome process. Especially, some F-box proteins were found to mediate ubiquitin-mediated proteolysis through WD-repeat domains, such as FBW8, FBXW2, beta-TrCP and F-box/WD repeat containing protein 7(FBW7).^47, 48, 49, 50, 51, 52, 53^ Moreover, some WD-repeat proteins were implicated in interaction with 20 different USPs in a proteomic screen.^54^ For example, WDR48 as well as two smaller homologues, USP12 and USP46, have recently been demonstrated to function as activators of USP1.^55, 56^ Due to its six individual WD-repeat domains, WDR79 belongs to the WD-repeat protein family. Previous studies show that WDR79 orchestrates the ubiquitin response, which is critical for DNA double strand break repair, as a scaffold protein by facilitating the interaction between E3 ligase RNF8 and its partner.^6^ Here we discovered a novel molecular function of WDR79: it is involved in the regulation of Mdm2 and p53 deubiquitination by interacting with USP7. Specifically, the deubiquitinating activity of USP7 depends on WDR79. The knockdown of WDR79 not only abolished the effect of USP7 on Mdm2 and p53 but also decreased the binding of USP7 to Mdm2 and p53. From a functional perspective, we speculate that, WDR79, a scaffold protein with multiple WD-repeat domains, may form a platform to actively recruit or tether USP7 and its targets Mdm2 and/or p53 from the nucleoplasm, which facilitates the USP7-mediated stabilization of Mdm2 and p53.

WDR79 mediates the stability of Mdm2 and p53 via USP7, but the latter two proteins execute opposing functions in various cellular settings.^42, 57^ This dichotomy raises an interesting question: how does WDR79 promote the proliferation of NSCLC cells? Based on our results, we found that the effect of WDR79 on Mdm2 is far stronger than that on p53. Because USP7 mainly deubiquitinates and stabilizes Mdm2 in unstressed cells, we believe that WDR79 may stimulate the USP7-Mdm2 axis more effectively than the USP7-p53 counterpart, thereby facilitating the proliferation of NSCLC cells despite the functional antagonism between Mdm2 and p53.

In summary, we have identified WDR79 as an upstream factor of USP7 and uncovered a new pathway from WDR79-USP7 interaction to growth promotion in NSCLC cells. Based on our results, we propose a model that accounts for the function of WDR79 via USP7 regulation. WDR79 may form a platform to recruit or tether USP7 and its targets Mdm2 and/or p53 from the nucleoplasm, which potentiates USP7 ability to deubiquitinate Mdm2 and p53, thereby stabilizing both Mdm2 and p53 and extending their half-lives. Given the opposite functions between p53 and Mdm2, we reason that the WDR79−USP7 interaction may render USP7 more active towards Mdm2 than p53, a dynamic interplay that ultimately results in the proliferation of NSCLC cells (Figure 7b). This view reinforces the importance of the relative effects on the Mdm2-p53 pathway in terms of their antagonism^43^ and links the complexity of additional factors to this central and competitive regulation. In conclusion, the role of the WDR79−USP7 interaction is a novel finding. Further investigations of this molecular process and the elucidation of its precise functions in the context of protein complex assembly and specific signal transmissions should yield novel insights into the activities of cancer-associated pathway.

## Materials and Methods

### Cell culture and reagents

Human NSCLC cell lines (H1299 and A549 cells) were cultured in RPMI-1640 medium (Gibco BRL Co. Ltd., Grand Island, NY, USA) with 10% fetal bovine serum (Gibco BRL Co. Ltd.) at 37 °C in 5% CO_2_ humidified incubators. Antibodies against p53 (DO-1), and Mdm2 (SMP14) were from Santa Cruz Biotechnology (CA, USA). The Mdm2 antibody (Ab-4) was from Millipore (Darmstadt, Germany), the USP7 and WDR79 antibodies were from Bethyl Laboratories (Montgomery, TX, USA), the GAPDH antibody was from KangChen Bio-tech Inc (Shanghai, China), and the antibody against ubiquitin (6C1.17) was from BD Pharmingen (Franklin Lakes, NJ, USA).

### Plasmids and transfection

To overexpress WDR79 or USP7 the full-length mRNA sequences were PCR-amplified from human cDNA and subcloned into pCMV-Tag2B to create Flag-tagged WDR79 or USP7 expression plasmid.

To stably knock down endogenous WDR79 in some cases, we used lentivirus-packaging shRNA expression vector (purchased from Gene Pharma, Shanghai, China) to infect cells. WDR79 shRNA target sequences were 5′-AATCAGCGCATCTACTTCGAT-3′. The control shRNA sequence was 5′-TTCTCCGAACGTGTCACGTTTC-3′.

### Immunoprecipitation assay

Immunoprecipitations were performed with Universal Magnetic Co-IP Kit (Active Motif, Carlsbad, CA, USA). First, 1 mg of crude extract was incubated with 3 μg of a relevant primary antibody or an isotype-matched negative control IgG overnight at 4 °C. Second, samples were incubated with 30 μl of magnetic beads conjugated with protein G19 for 1 h room temperature and washed three times with Co-IP/wash buffer. Next, precipitated proteins were dissolved in 30 μl 2 × SDS protein loading buffer, boiled for 10 min at 95 °C, and subjected to western blot analysis.

### Western blot assay

Following the aforementioned cellular treatments, cells were collected and lysed with M-PER buffer containing protease inhibitors (Pierce, Rock ford, IL, USA). Samples were resolved on 8–10% SDS-PAGE gels and transferred to nitrocellulose-membranes and blocked with 5% non-fat milk. Primary antibodies were incubated with the membranes overnight at 4 °C. Subsequently, membranes were washed and incubated with appropriate horse-radish peroxidase-conjugated secondary antibodies (Santa Cruz, CA, USA). Blots were developed with Chemiluminescence Detection Kit (Pierce).

### Indirect immunofluorescence assay

A549 and H1299 cells were fixed with 4% paraformaldehyde for 30 min, permeabilized with 0.2% Triton X-100 for 15 min, blocked with 5% bovine serum albumin, and then incubated with anti-WDR79 (Bethyl Laboratories, Inc., Montgomery, TX, USA), anti-p53, anti-MDM2 (Santa Cruz) or anti-USP7 (Bethyl Laboratories, Inc.) antibodies at 4 °C overnight, followed by a DyLight 594-conjugated or DyLight 488-conjugated secondary antibody (ImmunoReagents, Inc., Raleigh, NC, USA). Cells were stained with 2-(4-Amidinophenyl)-6-indolecarbamidinedihydrochloride (DAPI) (Beyotime Biotechnology, Haimen, China) for 10 min, and the images were acquired with a confocal-microscope.

### Protein half-life assay

Cells were treated with cycloheximide (50 μg/ml) for various periods to block protein synthesis. Crude extracts were prepared and protein levels were assessed by WB analysis.

### *In vivo* ubiquitination assay

Cells pretreated with 25 μM MG132 for 6 h were lysed and incubated with anti-Mdm2 or anti-p53 antibodies overnight at 4 °C. Then 30 μl beads conjugated with protein G were added and incubated for 1 h at room temperature. The immunoprecipitates were dissolved in 30 μl 2 × SDS protein loading buffer and analysed by WB using an anti-ubiquitin antibody.

### Cell proliferation assay

Cells were seed in 96-well culture plates, 3--(4, 5-dimethylthiazol-2-yl)-2, 5-diphenyltetrazolium bromide (Sigma, St. Louis, MO, USA) solution was added to each well at indicated time point and incubated at 37 °C for another 4 h. Supernatants were then aspirated, and the formazan product was dissolved with 100 μl dimethyl sulfoxide. The absorbance was measured at a wavelength of 570 nm with a microplate reader (Bio-Tek, Doraville, GA, USA).
